# Artificial Intelligence in Project Management: Challenges, Strategies and Best Practices

**DOI:** 10.12688/f1000research.169682.1

**Published:** 2025-12-04

**Authors:** Akinlo Mogbojuri, Obiyemi Obiseye, Arooj Wali, Mendon Dewa

**Affiliations:** 1 Department of Industrial Engineering, Durban University of Technology, Durban, KwaZulu-Natal, 4000, South Africa; 2 Space Science Centre, Dept. of Electrical Power Engineering, Durban University of Technology, Durban, KwaZulu-Natal, South Africa; 3 Faculty of Technology, University of Sunderland, Sunderland, England, UK

**Keywords:** Artificial Intelligence, Decision-Making, Scientometric approach, Qualitative analysis., Project Management

## Abstract

The application of Artificial Intelligence (AI) in project management is transforming decision-making processes, enhancing task execution, and improving risk management. This study aimed to elucidate the challenges raised by AI in project management (PM) using a scientometric and qualitative analysis. The research employs both quantitative and qualitative analysis using VOSviewer. The scientometric analyses reveal a substantial increase in AI in PM publications, with “project management,” “artificial intelligence,” “machine learning,” “cost reduction,” “decision making” and “supply chain management” as the most influential co-occurrence. The systematic review the implementation of challenges and strategies.

The analysis identifies the publication trends, most significant keywords, leading institutions and researchers, prominent collaboration connections, primary publication venues, and the most-cited publications. This research enhances understanding of AI in PM, promotes the utilization of artificial intelligence technologies for gaining insights during certain phases of project development, and improves project management efficiency. The utilization of AI technologies, including machine learning, natural language processing, and predictive analytics, markedly improves project efficiency by enhancing decision-making, effectiveness, and risk mitigation.

## 1. Introduction

Currently, artificial intelligence (AI) is a tangible phenomenon that is gaining significance. Nonetheless, other discoveries remain to be uncovered in both the scientific and commercial domains. The phrase artificial intelligence is very recent, emerging alongside the internet revolution. Markets and consumers have been anticipating these developments enabled by this new reality. Nonetheless, there exists opposition to its full implementation across numerous sectors ( Dwivedi et al., 2021). This poses a significant obstacle to the adoption of artificial intelligence and, in certain instances, has necessitated the establishment of regulations and guidelines to guarantee accountability and mutual respect among all players engaged ( Clarke, 2019; de Alcântara de Lima et al., 2022; Haenlein & Kaplan, 2019).

AI is progressively being incorporated into project management as firms endeavor to enhance efficiencies and improve decision-making for their future. Overseeing AI in project management (PM) is a multifaceted endeavor that necessitates recognizing diverse challenges, implementing effective strategies, and conforming to AI standards of excellence ( Tominc et al., 2024). Machine learning (ML), natural language processing (NLP), and predictive analytics have been recognized as significant domains in PM. The utilization of these tools has been shown to enhance decision-making, boost proficiency, and mitigate threats ( Kerzner, 2019).

A series of challenges accompany the incorporation of AI in PM. This indicates that a principal obstacle organizations face is reluctance to change. Employees may hesitate to adopt AI-powered technology due to social issues, such as fears of job loss or a lack of understanding regarding AI’s full potential. Moreover, insufficient data and data quality are significant obstacles, as AI necessitates substantial quantities of high-quality data for maximum performance ( Au-Yong-Oliveira et al., 2020). Moreover, AI methodologies necessitate intricate algorithmic system architectures; hence, the deployment of AI demands specific expertise and competencies, which are not consistently present in the existing workforce ( Jiang et al., 2021).

Proposals advocate for strategic efforts to address these difficulties utilizing. Although the utilization of AI presents some risks, organizational opposition can be mitigated by engaging stakeholders in the implementation process and providing comprehensive training courses ( Li et al., 2023). Integrating AI in PM is regarded as one of the brightest swings associated with enhanced resolution, increased efficiency, and effective project results. Nonetheless, this application is fraught with numerous problems that hinder its widespread adoption. Organizational inertia poses a challenge, since individuals may oppose change owing to potential job loss and a lack of faith in new AI-developed systems ( Miller, 2021). Furthermore, a significant skills deficit persists among project managers and colleagues, who frequently lack the technical expertise required to utilize AI technologies effectively ( Brougham & Haar, 2018). Another challenge in implementing AI is the standard or accessibility to data, as AI primarily relies on substantial volumes of high-quality data to guarantee the accuracy of its forecasts and analyses ( Wang, 2019). Nonetheless, the moral and accountability concerns surrounding algorithmic decisions hamper the advancement of the discipline, necessitating robust and proactive oversight ( Floridi et al., 2018). To address these challenges, comprehensive solutions are necessary, including training programs focused on AI and change management that delineate best practices for AI integration in PM, as well as ethical frameworks that govern the use of AI in PM contexts ( Paschen et al., 2020).

Thus, objectives of this study are as follows:

(1) To ascertain a principal issue that arises when employing AI in PM.
(2) Identify significant areas for enhancement concerning the incorporation of AI in PM.
(3) Perform a scientometric analysis of retrieved AI PM papers, including keywords co-occurrence analysis, research outlets contribution, science mapping of scholars, citations and contributions from organizations and countries.

## 2. Literature review

The combination of AI in PM has transformed traditional methodologies centered on the planning, implementation, and oversight of projects. The study examines the evolution of AI in project management, its transformations, and advantages for project enhancement.

### 2.1 Development of AI in PM

Niederman, (2021) has emphasized that the development of AI in PM has been remarkable, encompassing significant changes and notable enhancements in the planning, execution, and control of projects. Over time, AI tools have been integrated into PM processes, transforming them and generally improving project outcomes. Initially, AI was constrained in its applicability, mostly regarded as an academic subject and a theoretical idea ( Fridgeirsson et al., 2021).

Nevertheless, as computer power and data accessibility improved, AI applications emerged across several fields throughout the project’s duration, amongst is PM ( Trunk et al., 2020). Ong & Uddin, (2020) opined that the initial use of AI concentrated on decision-making systems that utilized project information/backgroud to provide knowledge, facilitating informed decision-making for project managers regarding forthcoming projects. These initial AI tools were crucial in enhancing project prediction and risk assessment. Similarly, Bento et al., (2022) asserted that as ML algorithms and NLP capabilities of AI advance, their role in projects has transitioned from observing to proactively managing increasingly repetitive and tedious work autonomously.

**Figure 1.**
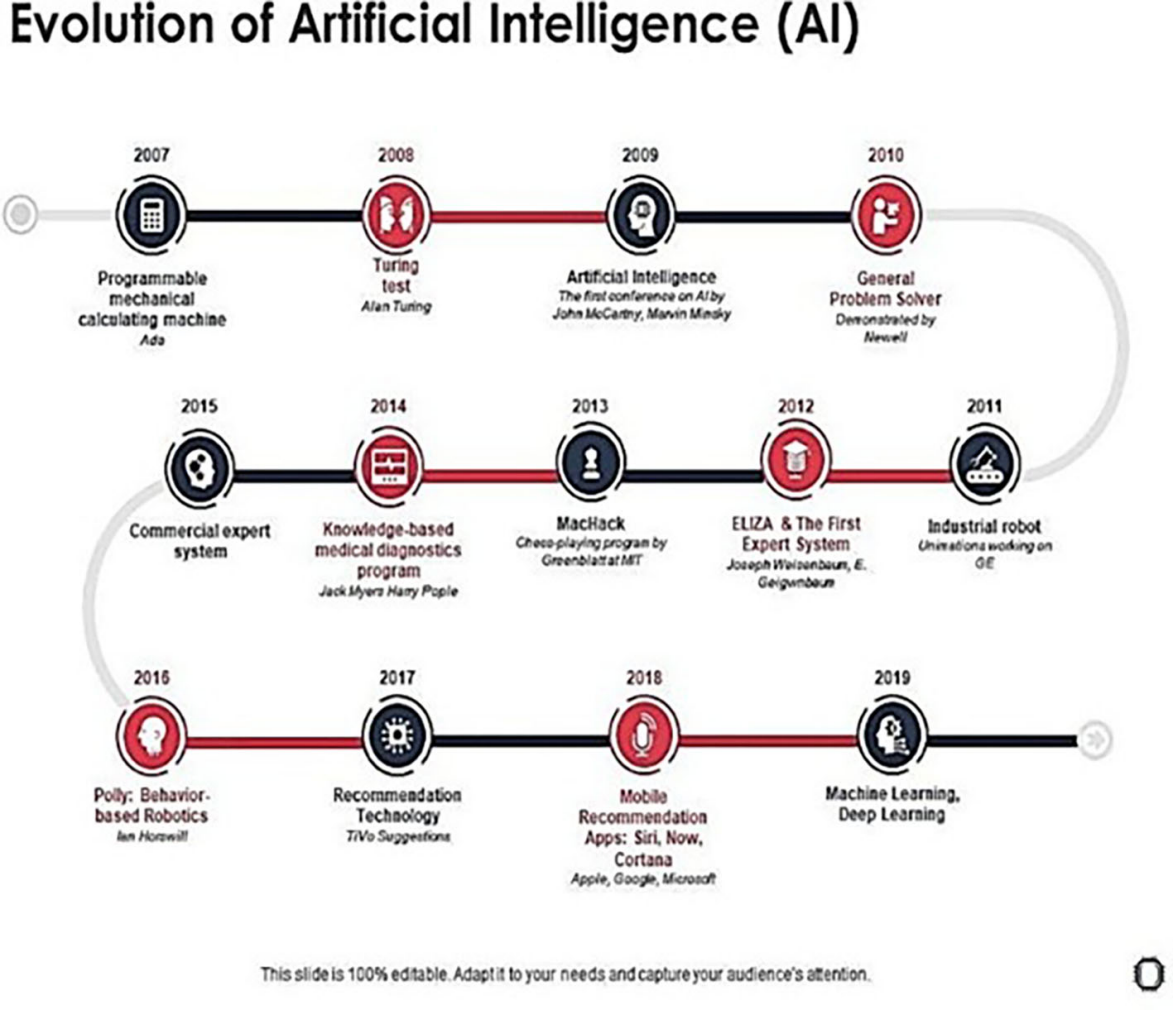
The evolution of artificial intelligence from 2007 to 2019 highlights significant milestones in programmable mechanical calculators, the Turing Test, general problem solvers, commercial expert systems, knowledge-based medical diagnostics, industrial robotics, behavior-based robotics, mobile recommendation applications, and advancements in machine and deep learning, featuring notable figures and companies such as Alan Turing, Newell, GE, Ian Horswill, Apple, Google, and Microsoft. Figure 1: ( Alshaikhi, 2021).

Study by Holzmann et al., (2022) asserted that the expansion of AI in PM will persist. Transformations such as AI-driven virtual assistants and enhanced combination with IoT systems will alter the PM procedure. AI has evolved from systems that support decisions to intellectual PM systems, enhancing project success, risk management, and resource optimization. The breakthroughs in AI are poised to revolutionize project management methods by integrating AI technology, hence aiding project managers and their teams in achieving substantial success in a swiftly changing corporate environment ( Ruiz et al., 2021). Recent studies suggest that integrating AI in PM represents a significant innovation trend, leveraging AI’s ability to oversee various operations, assess project performance, and facilitate important decision-making ( Li et al., 2023; Savio & Ali, 2023). The integration of artificial intelligence, particularly in ML, NLP, and robotics, is prevalent in the creation of project management software and related applications, as it enhances efficiency by reducing human errors, as noted in the available research ( Y. Pan & Zhang, 2021). Figure 1 shows the evolution of Artificial Intelligence (AI).

### 2.2 AI and PM

AI is a nascent concept employed across different fields. According to Singh & Haju, (2022), the transformations introduced by these innovations in project planning, execution, and management are groundbreaking. Nonetheless, there is a lack of systematic understanding of the extent and profundity of its influence across various areas. A separate study has demonstrated that AI may substantially improve PM tasks. AI solutions can assist project managers in enhancing decision-making, fostering collaboration among team members, mitigating risks, and improving the efficiency and efficacy of projects ( Bhbosale et al., 2020; Collins et al., 2021; Elrajoubi, 2020; Kunnathur, 2020; Munir, 2019). Furthermore, AI can mechanize monotonous tasks and consolidate data from various informants, thus allowing project managers to allocate time to other essential facets of PM ( Stamford, 2019; Xu et al., 2021).

### 2.3 Successful AI implementations in PM

**Figure 2.**
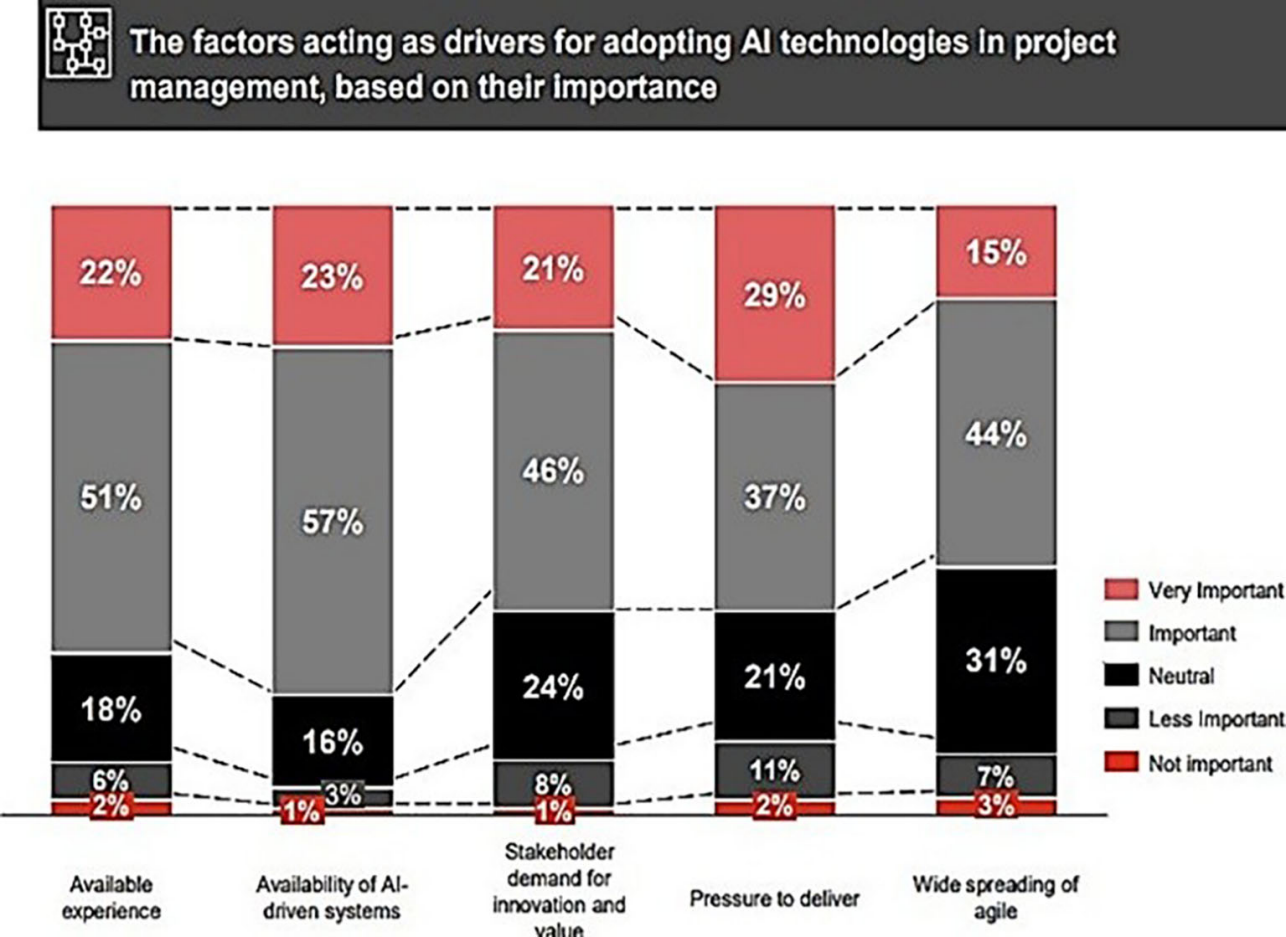
The elements driving the implementation of AI technologies in PM utilize a color-coding system to denote varying levels of significance: Red (top): very important; Gray: important; Black: neutral; Dark Gray/Black: less important; Red (bottom): not important. This illustrates five distinct factors: available experience, accessibility of AI-driven systems, stakeholder need for innovation and value, stress to produce, and the widespread use of agile methodologies. The Y-axis denotes the percentage breakdown (0-100%), with each stacked bar illustrating the percentage allocation of poll responses across the five degrees of importance for each factor. The percentages in each category reflect participants' evaluations of the significance of each aspect as a catalyst for AI adoption in PM.

The integration of AI technology in PM has demonstrated efficiency by transforming old procedures and enhancing project results ( Auth et al., 2021). Dam et al., (2019) demonstrated that intelligent systems in building projects are proficient in predicting project schedules, resource apportionment, and threat management procedures. By analyzing the accumulated data and reviewing the trends, AI models may forestall project restrictions, permitting project managers to implement appropriate actions to ensure projects are completed as scheduled. According to Sahadevan, (2023), AI has enhanced project resource management processes. It facilitates allocating appropriate resources to operations that must be executed at a designated time. Moreover, AI’s capacity to analyze extensive data sets allows it to discern hazards and evaluate their effects on project results ( Choi et al., 2022). Moreover, project managers receive support from virtual assistants utilizing artificial intelligence to perform regular administrative duties, coordinate meetings, and provide pertinent project information, facilitating more strategic decision-making for the project managers ( Salleh & Aziz, 2020). Using AI computational models for threat predicting and reduction can substantially enhance achievement rates ( Nahar et al., 2024; Shang et al., 2023). Figure 2, shows the factors propel the application of AI technologies in PM.

### 2.4 Challenges faced by organizations

**Figure 3.**
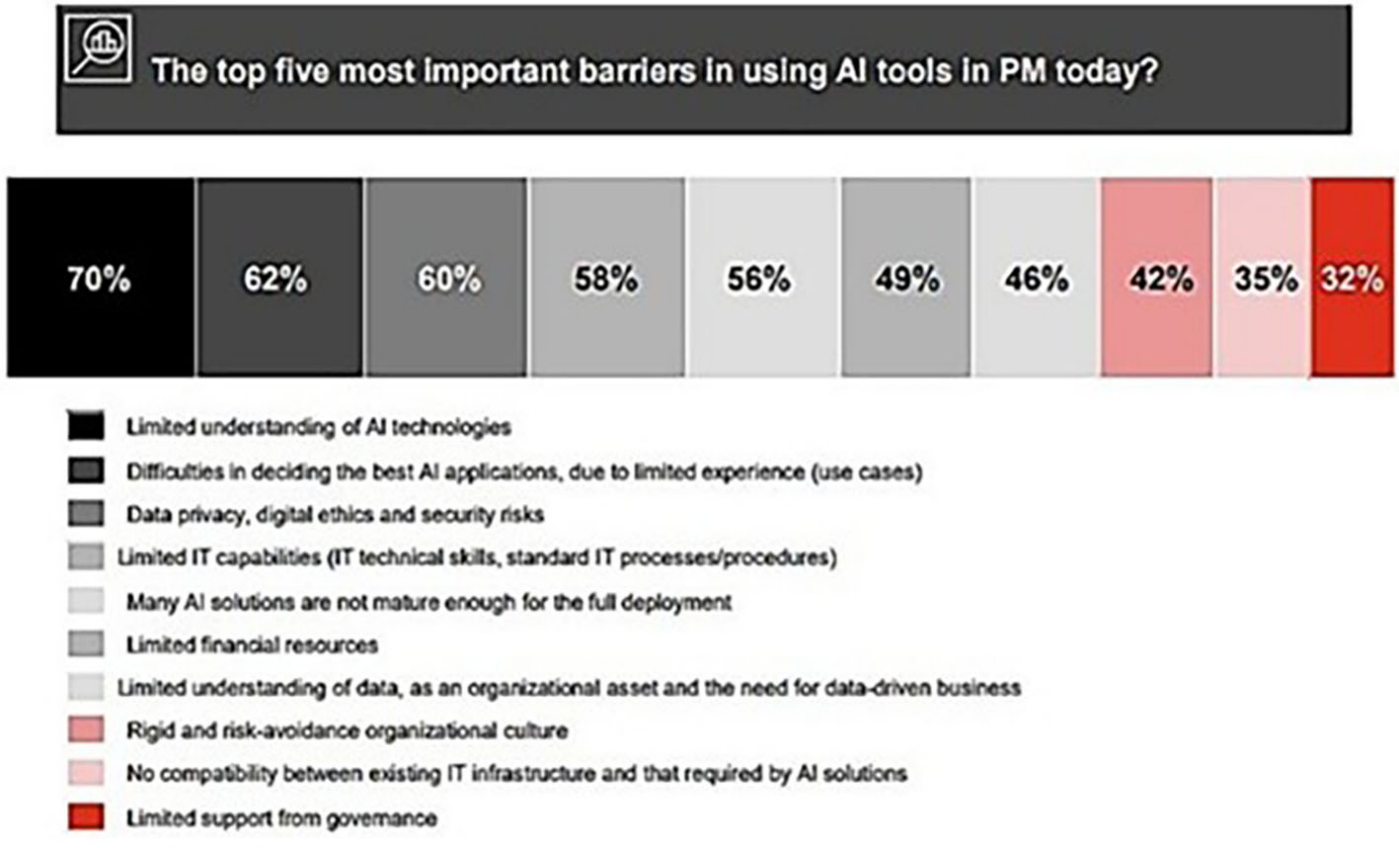
The five principal obstacles to the implementation of AI technologies in project management. Each hue, ranging from the darkest to the lightest, corresponds to a distinct barrier. 70% Black for Limited comprehension of AI technologies, 62% Dark Gray for Challenges in selecting optimal AI algorithms due to insufficient expertise (use cases), 60% Medium Gray for Data privacy, digital ethics, and security vulnerabilities. Light Gray indicates 58% of limited IT abilities (IT technical skills, conventional IT processes/procedures), whereas Very Light Gray signifies 56% for the immaturity of numerous AI solutions for complete adoption. Figure 3 **:** ( Bodea et al., 2020).

The use of AI in PM presents many obstacles for enterprises. These barriers can obstruct the efficient execution and employment of AI-driven PM systems ( Savio & Ali, 2023). Artificial intelligence relies heavily on superior and appropriate data to conduct accurate analyses and render informed recommendations. Numerous businesses encounter difficulties about the standard of their data, including issues of incompleteness, obsolescence, or inconsistency ( Kelepouris, 2023). Moreover, the process of extracting requisite information from several sources while ensuring data confidentiality might be complex. A further challenge that companies face is the uncertainty associated with initial capital outlay and profit. Incorporating AI into PM generally imply substantial advance fees for obtaining AI techniques, structure, and qualified individuals ( Thamhain, 2014). ( Davenport, 2018) observed that the expert’s shortage and workforce willingness to adopt AI continue to pose significant challenges for enterprises. AI integration must align with worker competencies in AI, data analysis, and ML. Challenges may emerge during the training of existing human resources or in the recruitment of AI professionals possessing pertinent expertise in a bound domain. Figure 3 shows barriers to the application of AI technologies in PM.

### 2.5 Best practices in AI implementation

Academic studies show that the utilization of AI and big data must adhere to established standards, as suggested by the subsequent best practices. Fountaine et al., (2019), assert the need of establishing communities to exchange best practices in AI and to expedite the integration of AI for value delivery. Davenport & Ronanki, (2018) emphasize the necessity for a measured first adoption and an aggressive acceleration plan to enable firms to optimize the advantages of AI tools. The study by Vandewinckele et al., (2020) outlines optimal strategies for integrating AI into radiotherapy workflows, including quality control measures. Strohm et al., (2020) identify the facilitators and obstacles of AI in radiology, emphasizing the importance of governmental guidelines and standards for optimal practice. The study by Magrabi et al., (2019) enhances the current discourse on the assessment of AI in clinical decision support by emphasizing the real-world implications of these procedures and highlighting the need to comprehend different types of AI alternatives.

### 2.6 Future development of AI in project management

Elrajoubi, (2020), investigate indicates that 56% of organizations have adopted a digital transformation plan incorporating Artificial Intelligence. By 2030, progressions in big data, ML, and NLP will allow Artificial Intelligence to oversee 80% of PM responsibilities. Diffendal, (2021) Recent research from project management institute, dubbed “Pulse of the Profession ^®^,” showed that more than eighty percent of participants indicated that AI influences their organization’s success. “Artificial Intelligence Entrepreneurs: Cracking the Code on Project Performance” suggests that project practitioners forestall an increase in the proportion of projects employing Artificial Intelligence from twenty-three percent to thirty-seven percent over the following three years of operation ( Cockburn et al., 2018).

**Figure 4.**
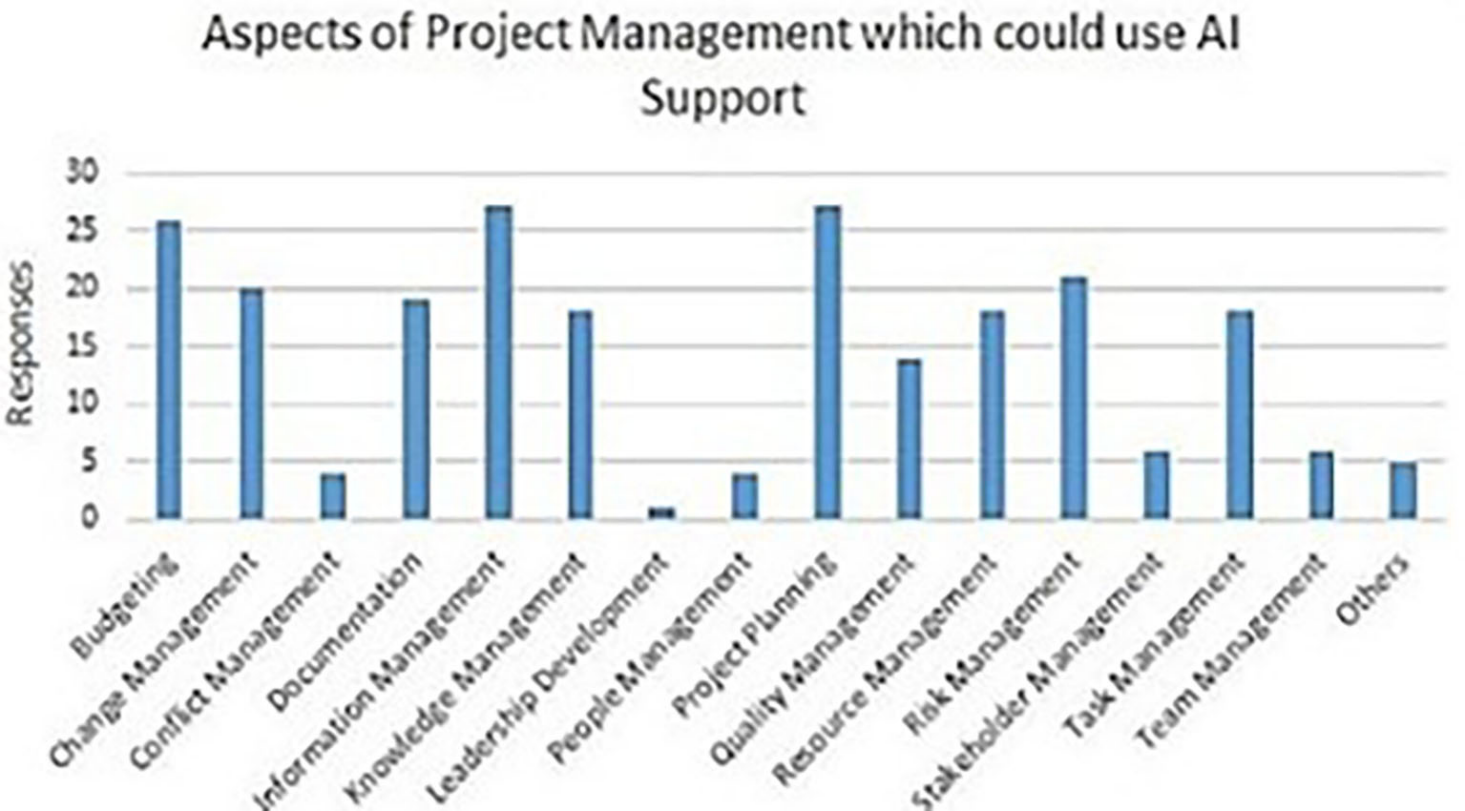
The facets of PM that potentially benefit from AI aid are shown by each bar, which delineates the specific area of PM deemed appropriate for AI assistance based on several survey results. The elevation of each bar directly correlates with the answer tally on the y-axis. Figure 4: ( Stamford, 2019) *.*

A current study by Markets and Markets projects that the AI market in PM will increase from two and a half billion United States dollars in 2023 to close to six billion United States dollars in the year 2028, reflecting a compound annual growth rate of 17.3 percent throughout the projection time ( Bezboruah & Bora, 2020). These findings suggest that AI drive increasingly influence the domain of PM in the coming years. Figure 4 delineates aspects of PM that may gain from AI support.

## 3. Methodology

**Figure 5.**
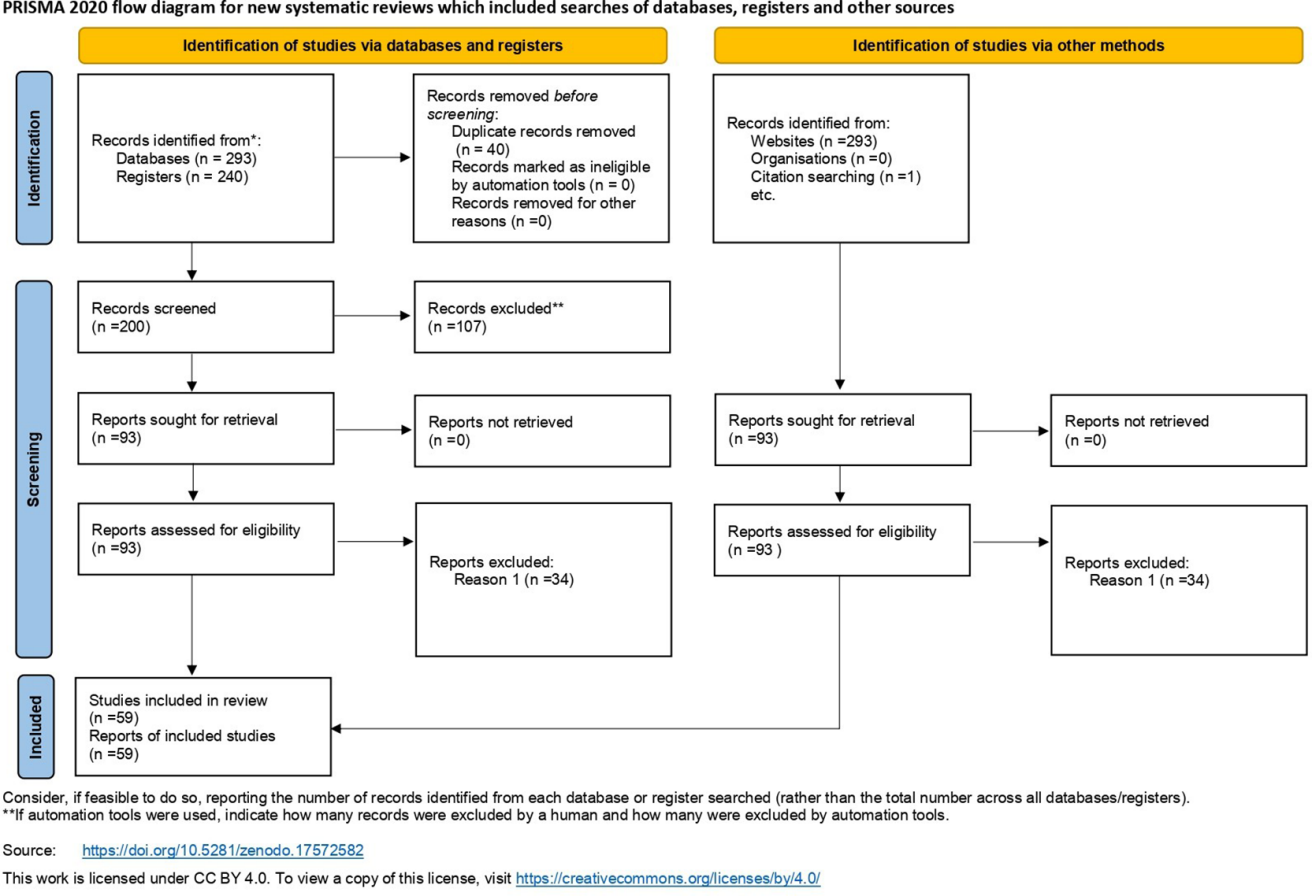
This graphic presents a PRISMA flow diagram delineating the systematic review procedure for selecting studies. The figure adheres to a conventional format featuring alternating green and red boxes linked by arrows, illustrating the transition from initial identification to final inclusion. The process commences with the identification of 240 records via data screening. After eliminating 40 duplicate entries, 200 records were left for evaluation. In the screening step, 107 records were removed from consideration, resulting in 93 full-text publications being evaluated for eligibility. After a comprehensive assessment of the full texts, an additional 34 publications were removed, leading to a total of 59 papers included in the systematic review.

**Figure 6.**
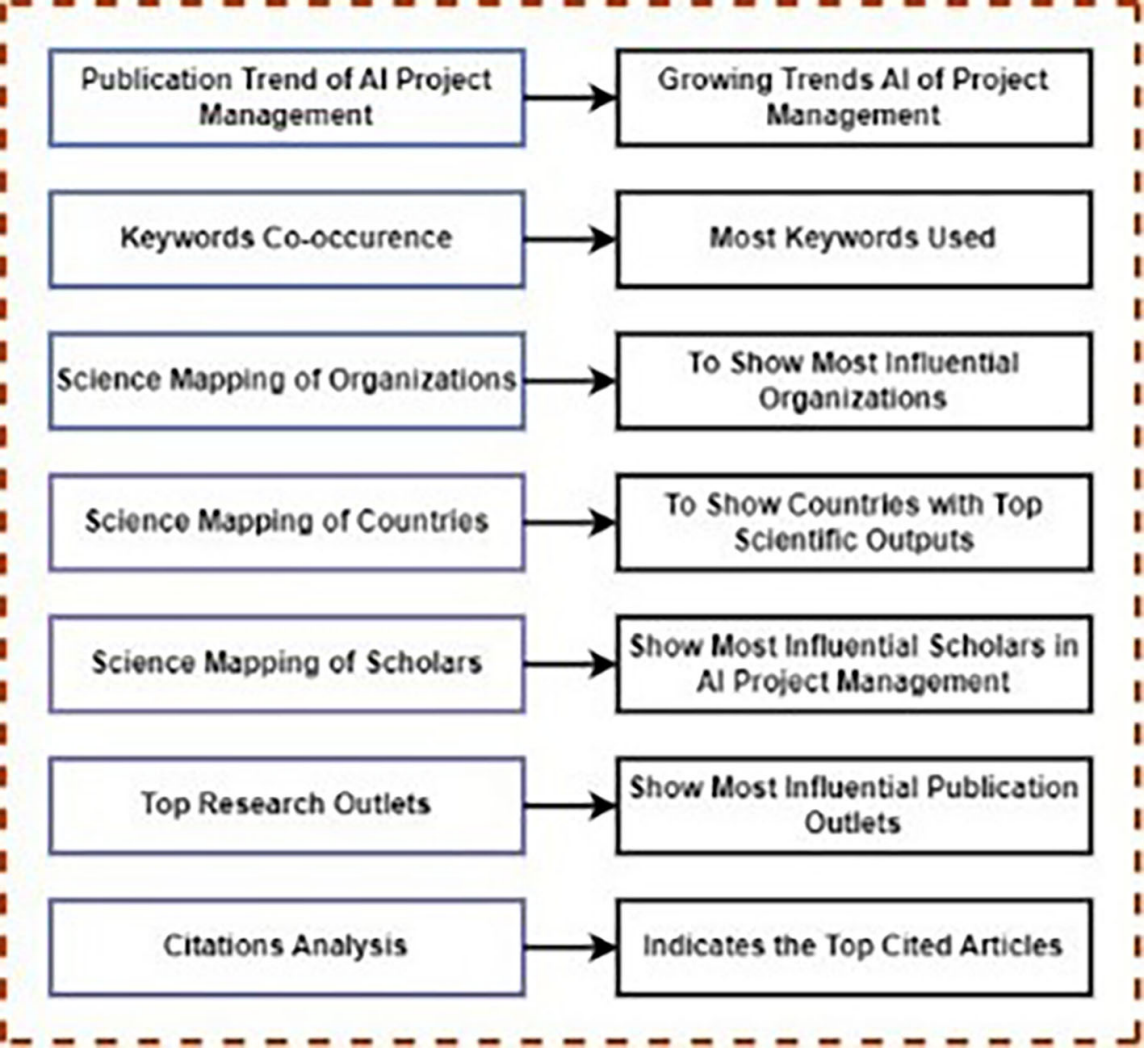
This framework offers a thorough bibliometric analysis structure, encompassing temporal trends, keyword patterns, institutional contributions, regional distribution, author influence, publication venues, and citation effect in the topic of AI in PM study.

The study utilized both qualitative and quantitative analysis which involves systematically searching for relevant literature and conducting scientometric analysis. Figure 5 provides a PRISMA flowchart of the procedures employed for literature retrieval. Additionally, Figure 6 illustrates the framework utilized in this study and outlining the process of conducting scientometric analysis. The following subsections provide further details on the methodologies employed.

### 3.1 Search method

The initial step entailed picking a suitable database, with Scopus being selected for its esteemed reputation for comprehensiveness ( Taiwo et al., 2023). A sequence of keyword evaluations and adjustments was performed to guarantee the discovery of pertinent studies. The keywords were meticulously selected to encapsulate the core of the research subject. Specifically, the TITLE-ABS-KEY field in Scopus was utilized, and the following keywords were adopted:

Keywords – [“Artificial Intelligence” OR “Project Management”] AND [“Challenges”] AND [“Strategies” OR “Best Practices”]. The selected keywords were meticulously designed to encompass the diverse facets of AI in PM and highlight the application of challenges or strategies in the inquiry.

The preliminary search produced 293 articles, which were subsequently improved by omitting non-English publications and those released prior to 2009, yielding 240 articles. Following the application of inclusion criteria (publication timeframe from 2009 to 2025, English language, journal articles, conference papers, reviews, and book chapters, with an emphasis on challenges and strategies for AI in PM), title and abstract screening resulted in the exclusion of 181 articles that predominantly addressed isolated techniques, yielding 59 pertinent publications. To guarantee thorough coverage, snowball sampling methods (both retrospective and prospective) were employed on the 43 articles, scrutinizing reference lists and monitoring citations to uncover more pertinent sources. This approach produced 16 additional articles, resulting in a total of 59 publications that constituted the foundation for analysis and synthesis in this study.

### 3.2 Scientometric analysis

Scientometric analyses employ scientific visualization, a technique developed by scholars for assessing bibliographic data across numerous fields. Scientometrics quantitatively evaluates scientific disciplines derived from published literature and communication. It encompasses identifying nascent scientific research domains, the analysis of research evolution across time, and the climatic and institutional distribution of research activities ( Mooghali et al., 2011). It attains many meanings by connecting articles, journals, authors, and keywords through co-citation and co-occurrence networks, which may facilitate potential developments and orientations ( Kanwar et al., 2023). The Scopus dataset was exported as Comma Separated Values (CSV) documents for processing with VOSviewer (version 1.6.20) software. VOSviewer is an open-source visualization software that is publicly accessible and extensively employed across various disciplines, with strong endorsements from scholars. The extracted bibliographic data (CSV) document was imported and evaluated with VOSviewer, ensuring data consistency and reliability ( Zhang et al., 2022). VOSviewer is a software application for scientific visualization ( McAllister et al., 2022). VOSviewer has been chosen for scientometric analysis in this study. This is due to their robust visualization capabilities, enabling researchers to examine and analyze intricate connections and interactions among texts, authors, and keywords ( Debrah et al., 2022).

### 3.3 Qualitative analysis

The qualitative study entails a comprehensive examination and synthesis of AI deployment in project management, comprising two parts. The initial part will tackle the implementation issues of AI in project management while also exploring practical solutions. The second phase will also focus on tactics for effective AI implementation in PM. The qualitative analysis will yield certain findings.

## 4. Results and discussion

This section presents the results of the scientometric analysis conducted in this study. This includes 1) publication trends, 2) keywords co-occurrence analysis, 3) science mapping of institutions, 4) science mapping of countries, 5) science mapping of scholars, 6) top research outputs, and 7) citation analysis.

### 4.1 Publication trends

**Figure 7.**
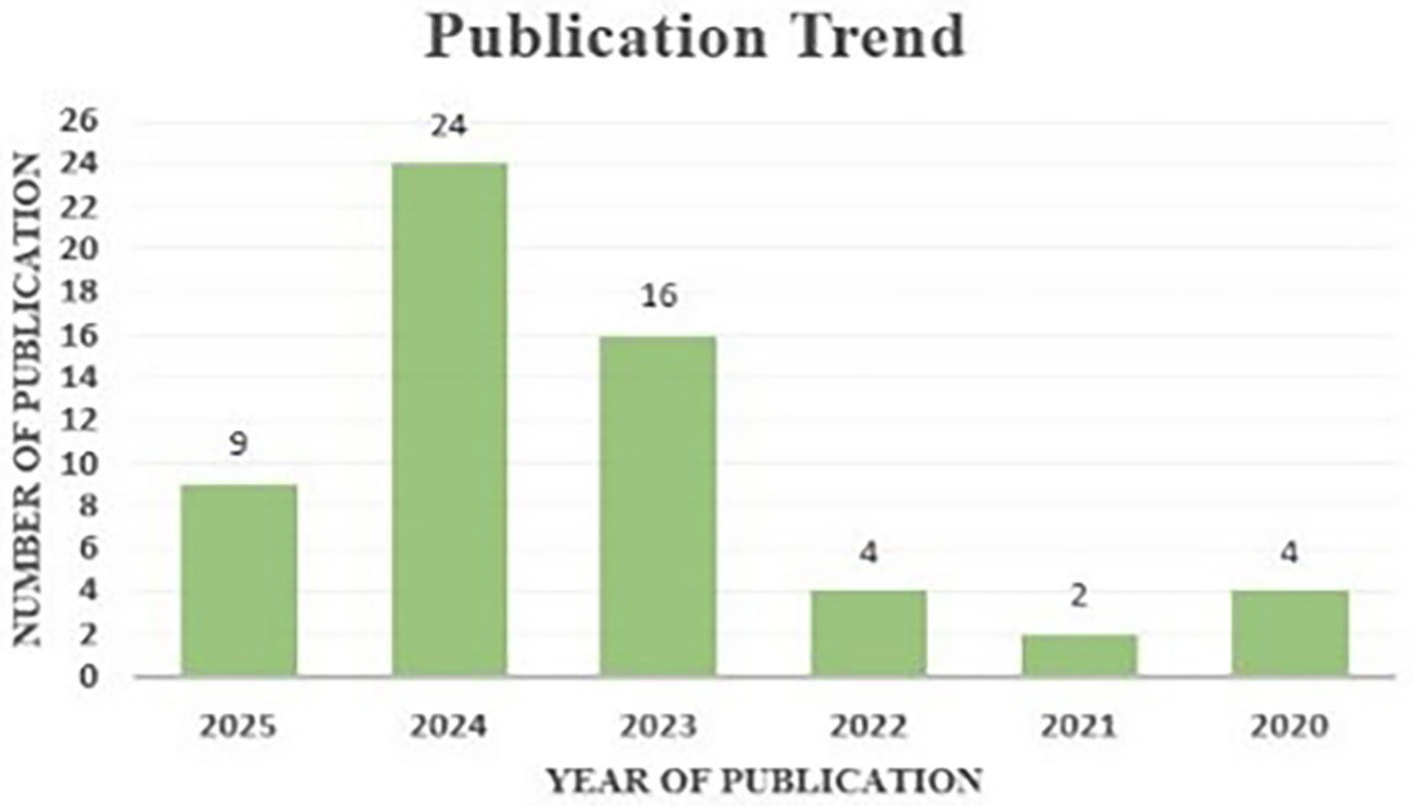
Publication patterns from 2020 to 2025 are represented, with each bar's height corresponding to the total number of publications, and exact figures indicated above each bar; 2024 has the most at 24 publications, while 2021 has the fewest at 2 articles.

The study of publishing patterns reveals a chronological view of the expansion and evolution of research in AI within project management. Figure 7 illustrates the annual publishing trend, demonstrating a consistent increase in the number of publications since 2022. This increased trend indicates the increasing interest and importance of AI in project management within academic and professional spheres. The findings on publishing trends highlight the escalating significance and visibility of AI in project management as a critical study domain, illustrating the mounting complexities and obstacles encountered by practitioners and scholars in effective project management. The changing environment of project management research offers potential breakthroughs and novel solutions for current project management challenges.

### 4.2 Keywords co-occurrence analysis

**Figure 8.**
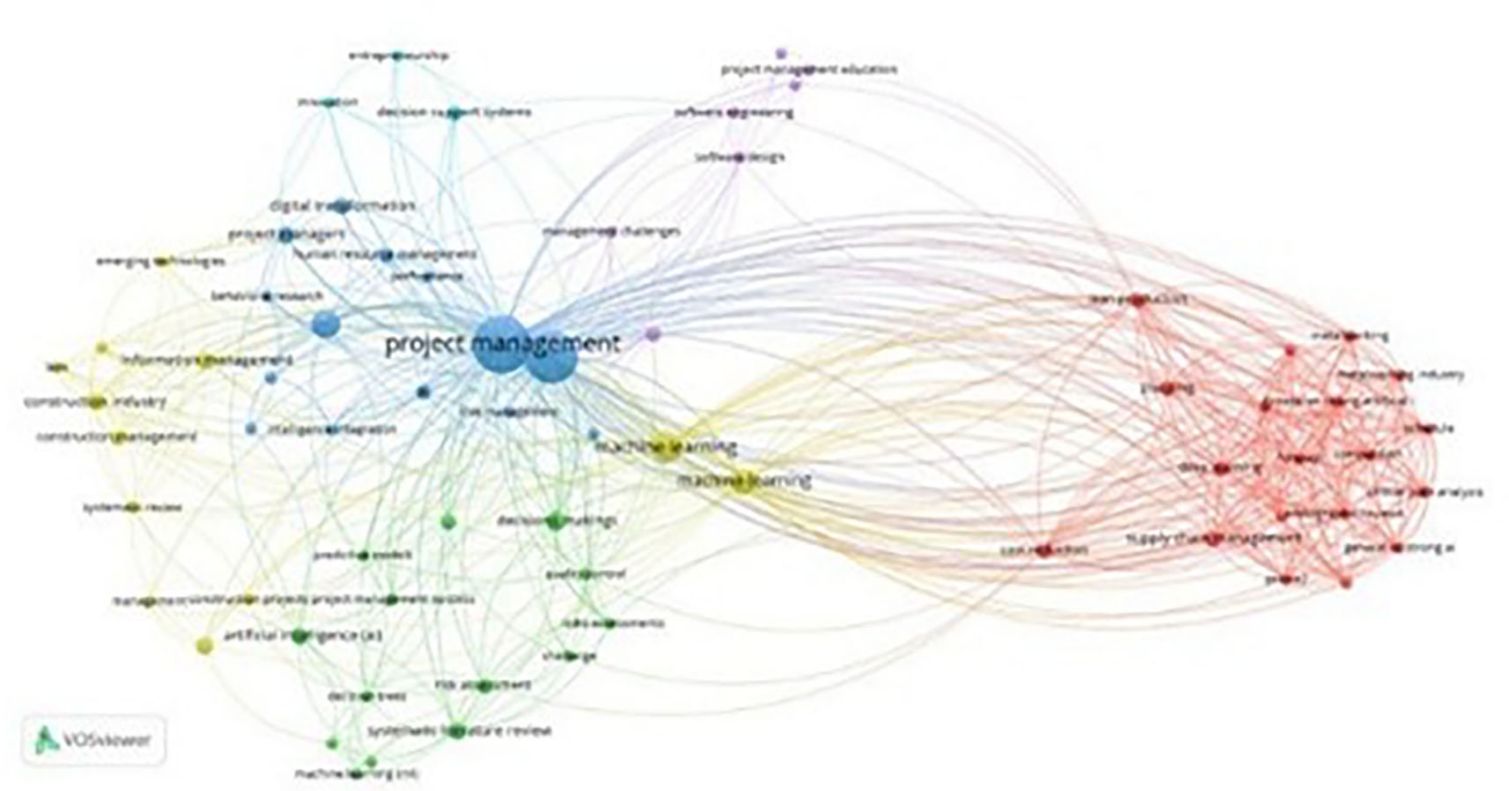
This is a co-occurrence analysis network map of keywords pertaining to AI in PM from 2009 to 2025, with distinct hues (red, yellow, green, blue, purple) denoting various theme groupings.

Co-occurrence analysis of keywords was performed utilizing VOSViewer software on 59 retrieved articles, revealing significant clusters of linked phrases and emphasizing key issues and trends in the field. The approach established a minimal occurrence threshold of 2, yielding 69 threshold keywords from an initial pool of 422 keywords. The network mapping categorized these terms into five unique color-coded clusters, with node size representing the rate of occurrence and inter-node length showing association strength in Figure 8. The keywords were ranked based on the total link strength (TLS) and occurrences shows “ PM,” “AI,” “ML,” “cost reduction,” “decision making” and “supply chain management” as the most influential terms in Table 1.

**Table 1.** Top 23 bibliometric analyses or keyword co-occurrence studies for AI in PM research domains.

Keywords	Occurrences	Total links strength
Project Management	43	192
Artificial Intelligence	36	163
Machine Learning	13	98
Cost reduction	3	52
Decision making	11	50
Supply chain management	4	50
Deep learning	3	48
Lean production	3	48
Planning	3	46
Bibliographic reviews	2	44
Competition	2	44
Critical path analysis	2	44
Forecast	2	44
Metalworking industry	2	44
Prince2	2	44
Resource valuation	2	44
Schedule	2	44
Supply chains	2	44
Decisions makings	5	27
Information management	4	25
Risk assessment	3	18
Systematic literature review	4	17
Project managers	4	17

### 4.3 Science mapping of organizations

**Figure 9.**
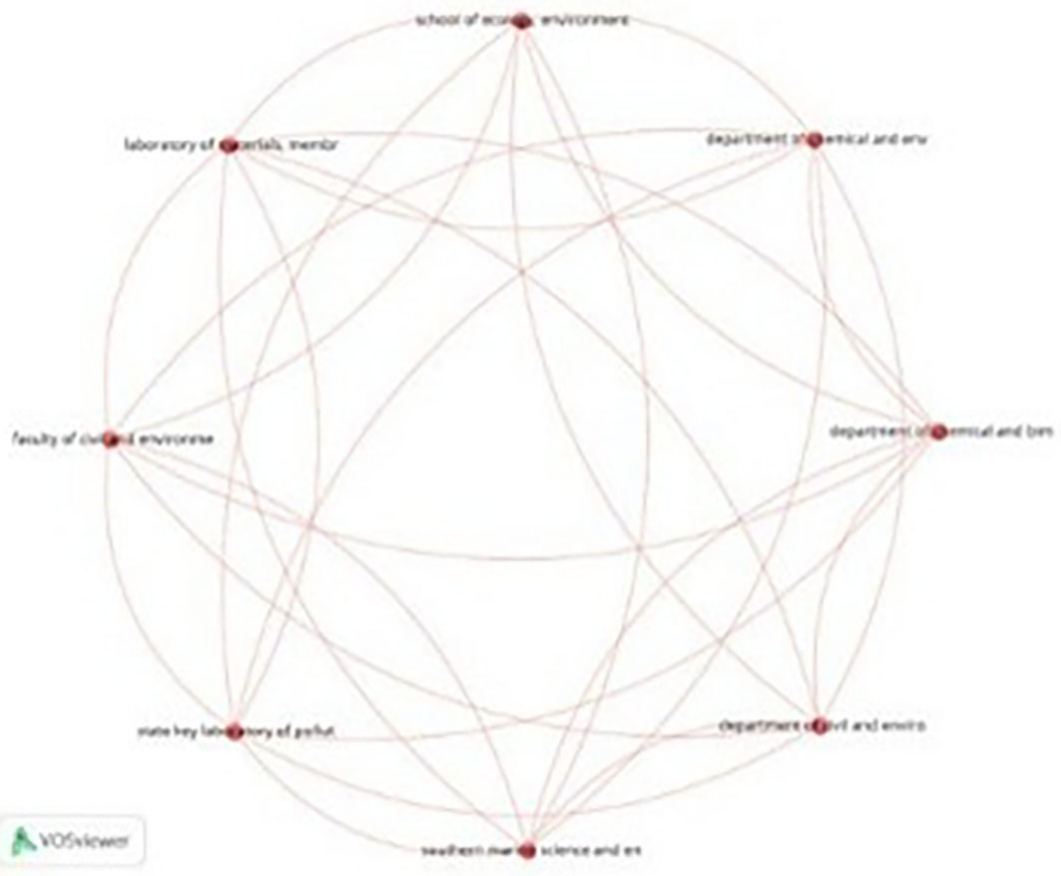
Prominent organizations Network mapping pertaining to the AI in project management research domain, featuring red lines that connect distinct nodes, illustrating relationships or partnerships among organizations.

The scientific mapping of organizations examined collaborative networks, co-authorship patterns, and research output among institutions in the subject of AI in project management. VOSViewer was utilized using the “co-authorship” analysis type, employing “organizations” as the unit of analysis, with exploratory thresholds established at a minimum of 1 document and 10 citations per organization. Out of 131 organizations in the collection, 27 institutions fulfilled these requirements, with nodes denoting institutions and arcs illustrating collaborative links in Figure 9.

Table 2 delineates the seven foremost institutions that have substantially contributed to the domain of AI in PM, ranked according to their TLS and citations. Vanderbilt University Nashville, Tennessee in United States, Yale University New Haven, Connecticut in United States, Technion — Israel Institute of Technology, Haifa in Isreal, University of Casablanca in Mohammedia in Morocco, University of Technology, Guangdong in China, Southern Marine Science and Engineering Guangdong Laboratory, Guangzhou in China and Tongji University, Shanghai in china are recognized as among the majority of prominent institutions in the area of AI in PM research. Their significant research output demonstrates their commitment to enhancing understanding in this domain. Universiti Tenaga Nasional, Kajang in Malaysia, and Imam Ja’afar Al-Sadiq University Baghdad in Iraq, lead in total link strength, indicating their robust involvement in joint research and alliances. The table comprises organisations from many geographical regions, notably the United States, Israel, Morocco, China, Malaysia, Iraq, and Australia, reflecting the global scope of AI in project management studies.

**Table 2.** The leading 16 organizations were rated according to their scholarly contributions to the discipline of AI in Project Management (PM).

Organizations	Document	Citations	Total links strength
Vanderbilt University Nashville Tennessee United States	1	36	7
Yale University New Haven Connecticut United States	1	36	7
Technion — Israel Institute of Technology, Haifa, Israel	1	36	7
University of Casablanca, Mohammedia, Morocco	1	36	7
University of Technology, Guangdong, China	1	36	7
Southern Marine Science and Engineering Guangdong Laboratory, Guangzhou, China	1	36	7
Tongji University, Shanghai, China	1	36	7
Universiti Tenaga Nasional, Kajang, Malaysia	1	24	3
Imam Ja’afar Al-Sadiq University, Baghdad, Iraq	1	24	3
Shanghai Jiao Tong University, Shanghai, China	1	11	2
University of Finance and Economics, Nanjing, China	1	11	2
Victoria University, Melbourne, Australia	1	53	1
University of the Basque Country—upv/ehu, Bilbao, Spain	1	53	1
University of Melbourne, Parkville, Australia	1	20	1
National University of Singapore, Singapore	1	20	1
University Putra Malaysia, Selangor, Serdang, Malaysia	1	16	1

### 4.4 Science mapping of countries

**Figure 10.**
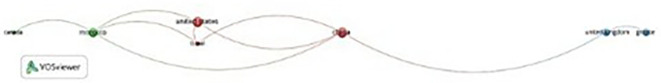
A network analysis of prominent nations about AI in PM development from 2009 to 2025.

Artificial intelligence in PM is a complex and extensively researched field that surpasses geographical limitations. The analysis of science mapping across countries provides a worldwide view on the research scene. This analysis facilitates a comprehensive comprehension of the contributions of various nations to AI in PM study, identifies global partnerships, and acknowledges the collective endeavors propelling developments in this domain globally. The VOSviewer software was utilized for the analysis type and the unit of analysis was designated as “co-authorship” and “countries,” accordingly. The minimum number of papers from a country was set to 1, and the minimum number of citations was also set to 1. Out of the 44 countries in the sample, 29 met the specified threshold criteria and selected 7 countries in Figure 10. Table 3 summarizes the 13 prominent countries in AI project management research, detailing their respective document counts, citation totals, and overall link strength. The table is sorted according to TLS, positioning China, Morocco, and the United States at the forefront, strengthening their collaborative ties within the AI in PM research community. Regarding research efficiency, China and Morocco are the most prolific nations, although Australia and China exhibit the highest citation impact. These outcomes underscore in furthering knowledge and comprehension in the domain of AI in PM. The global presence encompasses continents such as Asia, Africa, North America, Europe, and Oceania, underscoring the international interest and advancement in artificial intelligence technologies for project management.

**Table 3.** The 13 countries are ranked according to their impact and contributions to AI in project management research.

Countries	Document	Citations	Total links strength
China	5	56	4
Morocco	4	41	4
United States	5	48	3
Israel	1	36	3
Iraq	2	24	3
Jordan	3	16	3
Australia	2	73	2
Malaysia	3	40	2
United Kingdom	3	8	2
Germany	4	6	2
India	4	5	2
Spain	3	55	1
Greece	3	24	1

### 4.5 Science mapping of scholars

**Figure 11.**
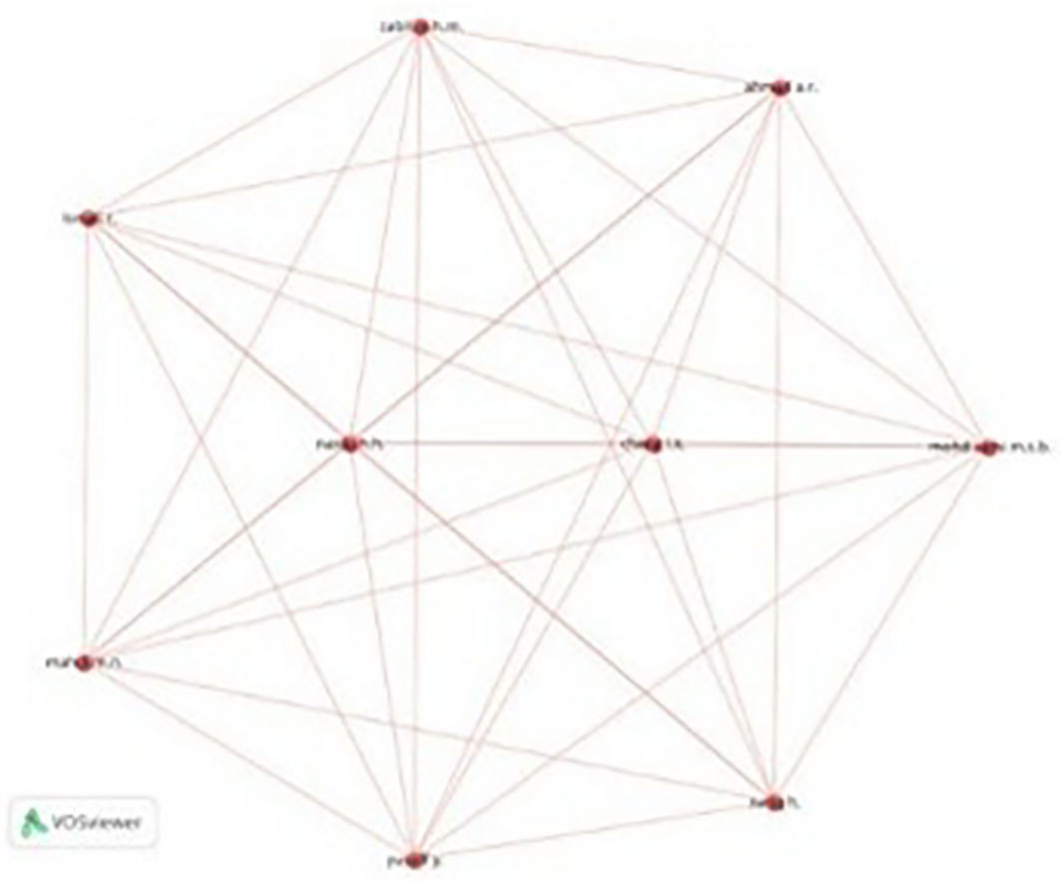
Co-authorship or collaboration network among researchers and academics publishing on AI in the project management research domain from 2009 to 2025.

This investigation explores the intricate network of researchers, revealing collaboration practices and the distribution of knowledge within the area of AI in PM. The analysis identifies prolific writers and renowned research groups that have significantly contributed to the growth of knowledge in AI within project management. The analysis type and unit in VOSViewer were designated as “citation,” “author,” and “total link strength,” accordingly. The maximum number of authors and citations was established at 1 and 10, respectively. Among the 195 writers identified in the 59 publications, 41 researchers fulfilled these requirements. Figure 11 illustrates the visual correlation among the authors. Table 4 delineates the ten foremost research academics in the area of AI in PM, as determined by the data utilized for the investigation. The ranking employs TLS, positioning “Del Cerro M.,” “Elimelech M.,” and “Epsztein R.” at the top.

**Table 4.** Leading/Top 18 researchers in the research area exhibiting robust inter-author interactions and collaboration connections within the topic of AI in PM.

Authors	Document	Citations	Total links strength
Del Cerro M.	1	36	8
Elimelech M.	1	36	8
Epsztein R.	1	36	8
Lin S.	1	36	8
Liu W.	1	36	8
Livingston J.L.	1	36	8
Wang L.	1	36	8
Wang Z.	1	36	8
Younssi S.A.	1	36	8
Yusoff Y.	1	24	8
Ahmad A.R.	1	24	8
Cheng L.K.	1	24	8
Ismail R.	1	24	8
Mahdi M.N.	1	24	8
Mohd Azmi M.S.B.	1	24	8
Naidu H.H.	1	24	8
Natiq H.	1	24	8
Zabil M.H.M.	1	24	8

### 4.6 Top research outlets

This section presents the premier research outlets in the field of AI in PM. Finding credible and influential publication outlets is essential for comprehending the primary sources propelling improvements in the field and for obtaining high-quality research that directs scholars, professionals, and followers to reliable sources for the most recent findings, approaches, and conceptual developments. It can also aid educational institutions in choosing appropriate journals to optimize their investment strategy. Utilizing VOSViewer, the minimum threshold for documents and citations was established at 1 and 3, respectively, fulfilling the criteria for 21 out of 53 research outlets. Table 5 enumerates the nine foremost research outlets in the AI in PM domain according to the TLS, with the leading three publication outlets being “Journal of Open Innovation: Technology, Market, and Complexity,” “Built Environment Project and Asset Management,” and “IEEE Engineering Management Review.” This breakdown reflects a varied publishing environment, featuring both significant niche journals and interconnected multidisciplinary platforms that contribute to AI in PM research.

**Table 5.** Premier research portals that demonstrate differing degrees of research influence and interconnectivity among 9 academic publications in the subject of AI in PM research.

Research outlets	Documents	Citations	Total links strength
Journal of Open Innovation: Technology, Market, and Complexity	1	3	7
Built Environment Project and Asset Management	1	20	2
IEEE Engineering Management Review	2	12	2
Applied Sciences (Switzerland)	2	77	1
Journal of Modern Project Management	1	20	1
Asian Journal of Civil Engineering	1	16	1
International Journal of Advanced Computer Science and Applications	2	12	1
Management Review Quarterly	1	10	1
Journal of Theoretical and Applied Information Technology	2	8	1

### 4.7 Citation analysis

Citation analysis intended to illuminate the most frequently cited works in the field of AI in project management. Comprehending the significant works and their effects on the research community is crucial for research scholars, academics, and practitioners. Thirteen mentioned papers are a curated collection of works that have significantly influenced the field of AI in project management, affecting its theoretical underpinnings, methodological frameworks, and practical implementations. Numerous factors, including the citation culture of the discipline, the age of publications, and the general magnitude of the research community, might affect citation numbers. Table 6 displays the ten most-cited works in the domain of AI in PM, offering essential information such citation count, TLS, and reference data. The study by Taboada et al. (2023) on Artificial Intelligence Enabled Project Management: A Systematic Literature Review has attracted considerable interest from researchers. The publishing years span from 2020 to 2024, signifying a dynamic and swiftly advancing research domain.

**Table 6.** Ranking of the top 10 important papers based on citation influence in the field of AI in project management.

Titles	Citations	Total links strength	References
Artificial Intelligence Enabled Project Management: A Systematic Literature Review	53	2	( Taboada et al., 2023)
Pressure-Driven Membrane Desalination	38	2	( Liu et al., 2024)
Prospects, Drivers of And Barriers to Artificial Intelligence Adoption in Project Management	21	2	( Shang et al., 2023)
The Role of Artificial Intelligence in Project Management	12	2	( Odeh, 2023)
Towards A Hybrid Project Management Framework: A Systematic Literature Review on Traditional, Agile and Hybrid Techniques	21	1	( Papadakis & Tsironis, 2020)
Applying Machine Learning and Particle Swarm Optimization for Predictive Modeling and Cost Optimization in Construction Project Management	16	1	( Almahameed & Bisharah, 2024)
Big Data, Data Science, and Artificial Intelligence for Project Management in The Architecture, Engineering, And Construction Industry: A Systematic Review	12	1	( Zabala-Vargas et al., 2023)
Project Management in the Fourth Industrial Revolution	12	1	( Cabeças & Da Silva, 2020)
A Systematic Review of the Knowledge Domain of Institutional Theory in Construction Project Management	11	1	( Qiu & Chen, 2023)
How Artificial Intelligence will Transform Project Management in the Age of Digitization: A Systematic Literature Review	10	1	( Nenni et al., 2024)
Artificial Intelligence in Project Management Research: A Bibliometric Analysis	8	1	( Maphosa & Maphosa, 2022)
Exploring The Challenges and Impacts of Artificial Intelligence Implementation in Project Management: A Systematic Literature Review	7	1	( Hashfi & Raharjo, 2023)
Artificial Intelligence in Open Innovation Project Management: A Systematic Literature Review on Technologies, Applications, And Integration Requirements	3	9	( Prasetyo et al., 2025)

### 4.8 Results of the qualitative analysis

This section delineates the results of the systematic review of current studies about the defiance and strategies related with AI application in the context of PM research.

**4.8.1 Overcoming implementation challenges**

A research analysis examining techniques to tackle the implementation issues of AI in PM also explored feasible options. Bharati & Sandbrink, (2024) examine the effect of AI on PM, emphasizing the challenges associated with AI integration. El Khatib & Al-Sadi, (2023) underscore the obstacles associated with the deployment of smart solutions in the integration of IoT into projects. McGrath & Kostalova, (2020) examine trends and disturbances in PM post-2020, focusing on AI and robotics. Brock & Von Wangenheim, (2019) examine the potential and actual challenges of surmounting obstacles to AI approval and the elements contributing to company achievement. Drmac, (2022) delineates the steps that a project manager can implement to eradicate obstacles to the deployment of AI. Hashfi & Raharjo, (2023) systematic literature review extensively examines the obstacles associated with implementing AI in PM situations. Mohammad & Chirchir, (2024), examine the primary problems associated with the implementation of AI in the project planning phase. The research by Bérubé et al., (2021) identifies the organizational hurdles to adoption based on the findings of the Delphi survey conducted by ( Tominc et al., 2023). They implemented agile project management with artificial intelligence through the formulation of an equation and mitigation methods. Singh & Haju, (2022) primarily focuses on the challenges associated with the combination of PM offices. Ångström et al., (2023) offer insights from a global survey of enterprises regarding AI deployment. Hedlund & Henriksson, (2023) investigate challenges related to the implementation of Responsible AI suggestions.

**4.8.2 Strategies for successful AI integration**

Numerous researches examine strategies to improve AI integration in PM and identify optimal procedures in the field. According to McGrath & Kostalova, (2020), project management trends encompass digitalization and the exploration of artificial intelligence applications in PM. Dzhusupova et al., (2024) present a strategic management framework for the integration of AI in engineering sectors, highlighting potential risks and optimal mitigation strategies. W. Pan & Zhang, (2023) have elucidated the present state and prospective developments of integrating BIM and AI for intelligent construction management. A study by Dad et al., (2024), examines the influence of AI, team ability, and institutional backing on enhancing PM success rates in Pakistan. Regona et al., (2023) previously examined the literature about AI deployment in the construction sector, focusing on the associated potential and difficulties. In the study of Brock & Von Wangenheim, (2019) they elucidates how corporations might deploy AI, incorporating insights from companies which have experienced digitization. Hanafi et al., (2022) elucidate the selection of project managers utilizing artificial intelligence, actively integrating emotional intelligence with informatics.

## 5. Conclusions

This study was driven by the necessity to perform a scientometric analysis and qualitative assessment primarily centered on AI in PM. As PM methods become increasingly sophisticated in the contemporary period, there is a critical necessity to utilize artificial intelligence. The research sought to identify the fundamental concerns associated with the use of AI in project management, ascertain potential significant areas for enhancement, and conduct a scientometric analysis. The aims were accomplished through a rigorous process that included a systematic literature search, quantitative scientometric analysis with VOSviewer, and a qualitative examination of 59 articles obtained from the Scopus repository. The study concludes as follows:

(1) The annual publishing trends which demonstrates a consistent increase in the number of publications since 2022.
(2) The “PM,” “AI,” “ML,” “cost reduction,” “decision making” and “supply chain management” as the most influential keywords within the AI in PM.
(3) The seven foremost institutions that have substantially contributed to the domain of AI in PM.
(4) According to TLS, positioning China, Morocco, and the United States at the forefront, strengthening their collaborative ties within the AI in PM research community.
(5) Positioning “Del Cerro M.,” “Elimelech M.,” and “Epsztein R.” are the foremost research academics in the area of AI in PM.
(6) Shown three most publication outlets being “Journal of Open Innovation: Technology, Market, and Complexity,” “Journal of Open Innovation: Technology, Market, and Complexity,” and “IEEE Engineering Management Review.”
(7) Presented most-cited works in the domain of AI in PM such as “Artificial Intelligence Enabled Project Management: A Systematic Literature Review.”

The combination of AI in PM improves its effectiveness and efficacy in decision-making and project achievement. Artificial intelligence tools facilitate the acquisition of insights throughout certain phases of a project’s development, prompt identification of disruptive forces, streamline routine tasks, and improve project management efficiency. The long-term benefits of AI indicate its potential to significantly enhance project management, particularly regarding sustainability and stakeholder engagement. The research indicated that the utilization of AI, encompassing ML, NLP, and predictive analytics, significantly enhances project performance by improving decision-making, efficiency, and risk mitigation.

The recognized best practices for AI integration in PM emphasize the necessity for refinement via iterative evaluation and verification of AI models, alongside adherence to moral standards to prevent adverse effects such as bias and discrimination, ensuring fairness in implementation. AI can enhance PM by increasing efficiency and effectiveness, thereby leading to increased stakeholder satisfaction.

The study has some limitations.

(1) Publishing prejudice may arise from utilizing various databases in the systematic literature review.
(2) Diverse AI technologies and PM approaches are employed across various sectors and regions.
(3) Certain facets of AI technologies and their uses are ephemeral, and forthcoming improvements may alter the current information.
(4) It is essential to recognize that AI and PM encompass various fields, requiring professionals to possess diverse skill sets.

Additionally, subsequent study should concentrate on delineating the protocols and methodologies employed for AI integration in various project management contexts. Such frameworks can aid organizations in formulating the most effective strategies for AI utilization, considering the particularities of diverse sectors and initiatives. Consequently, the rapid evolution of AI technology necessitates ongoing research to remain abreast of emerging innovations. This entails performing a literature review to identify emerging technologies associated with AI and their potential use in project management to maintain the relevance of the research issue.

## Data Availability

Zenodo. Artificial Intelligence in Project Management: Challenges, Strategies and Best Practices. https://zenodo.org/records/17668834 This project contains the following underlying data:
• Scientific Framework. (Includes publication trends, keywords co-occurrence, science mappings for organization, countries and scholars, top research outlet and citation analysis) • PRISMA flowchart. (240 records identified, 200 records duplicates removed, 107 records excluded, 93 full text articles assessed, 34 full texts excluded and 59 studies included in systematic review) • Checklist. (Checklist item includes title and abstract, introduction, method, results, discussion, conclusion and other information’s) Data are available under the terms of the Creative Commons Attribution 4.0 International license (CC-BY 4.0).
