# Supplementary material for: Artificial Intelligence in Project Management: Challenges, Strategies and Best Practices
Source: F1000Res. 2026 Apr 15;14:1357. Originally published 2025 Dec 4. [Version 2] doi: 10.12688/f1000research.169682.2 (PMC13215357; doi:10.12688/f1000research.169682.2)
Supplement: Peer review history

# Reviewer report: Reviewer response for version 2

**DOI:** 10.5256/f1000research.197662.r475293

**Published:** 2026-04-29

**Authors:** Satyadhar Joshi

**Affiliations:** 1 Assistant Vice President, Global Analytics, Bank of America, Jersey City, NJ, USA

Paper looks much better, no further comments.

Are the rationale for, and objectives of, the Systematic Review clearly stated?

Yes

Is the statistical analysis and its interpretation appropriate?

Partly

If this is a Living Systematic Review, is the ‘living’ method appropriate and is the search schedule clearly defined and justified? (‘Living Systematic Review’ or a variation of this term should be included in the title.)

Partly

Are sufficient details of the methods and analysis provided to allow replication by others?

Partly

Are the conclusions drawn adequately supported by the results presented in the review?

Partly

Reviewer Expertise:

Agentic AI, Generative AI, AI risk, AI Management, Risk Management, Business Management

I confirm that I have read this submission and believe that I have an appropriate level of expertise to confirm that it is of an acceptable scientific standard.

# Reviewer report: Reviewer response for version 1

**DOI:** 10.5256/f1000research.187040.r461775

**Published:** 2026-03-18

**Authors:** Ogochukwu Gold Abaneme

**Affiliations:** 1 Northeastern University, Boston, USA

The article highlights the application of AI in project management, including successful implementation and challenges. The article included a scientometric analysis of existing papers on AI in PM and including the universities and countries where the papers originate from. Through the analysis we have been able to see an increase in publication on AI in PM and major keywords being used. However, the article does not necessarily highlight areas in PM the identified publication discusses and gaps for further research. This is not necessarily an issue because project management is wide and applicable in different fields and has been identified in this articles as a limitation.

 Some information needed to be corrected includes the following: 

 A. "2.6 -  Diffendal, (2021) Recent research from project management institute, dubbed “Pulse of the Profession®,” showed that more than eighty percent of participants indicated that AI influences their organization’s success. “Artificial Intelligence Entrepreneurs: Cracking the Code on Project Performance” suggests that project practitioners forestall an increase in the proportion of projects employing Artificial Intelligence from twenty-three percent to thirty-seven percent over the following three years of operation (Cockburn et al., 2018)".

      the first two lines of this paragraph is confusing. are you quoting Diffendal's writing on pulse of the profession, if so, the 'Recent' should start with a small letter. however, it is advisable to quote pulse of the profession directly for easier reference.

 "Figure 5 - This graphic presents a PRISMA flow diagram delineating the systematic review procedure for selecting studies. The figure adheres to a conventional format featuring alternating green and red boxes linked by arrows"

 **i see yellow and blue boxes** not purple and green

 Figure 8 - the ppt slide is blurry and the only words I was able to deduce was Project management. It would be best to find a way to ensure that the figure is clear to your readers.  (I also downloaded the ppt and had the same issue)

 Figure 9 10 & 11 has same issue

 Table 6 is a well detailed table; however it contains 13 papers, not 10 as stated. you can either remove 3 or change 10 to 13.

  In the study of Brock & Von Wangenheim, (2019) they elucidate - replace off with by and elucidates with elucidate.

 Once corrected, I believe the article can be approved

Are the rationale for, and objectives of, the Systematic Review clearly stated?

Yes

Is the statistical analysis and its interpretation appropriate?

Yes

If this is a Living Systematic Review, is the ‘living’ method appropriate and is the search schedule clearly defined and justified? (‘Living Systematic Review’ or a variation of this term should be included in the title.)

Not applicable

Are sufficient details of the methods and analysis provided to allow replication by others?

Yes

Are the conclusions drawn adequately supported by the results presented in the review?

Yes

Reviewer Expertise:

Project Management and AI

I confirm that I have read this submission and believe that I have an appropriate level of expertise to confirm that it is of an acceptable scientific standard, however I have significant reservations, as outlined above.

# Response

**Published:** 2026-04-16

**Authors:** Akinlo Mogbojuri

**Affiliations:** Industrial Engineering, Durban University of Technology, Durban, KwaZulu-Natal, South Africa

**Comment**

 The article highlights the application of AI in project management, including successful implementation and challenges. The article included a scientometric analysis of existing papers on AI in PM and including the universities and countries where the papers originate from. Through the analysis we have been able to see an increase in publication on AI in PM and major keywords being used. However, the article does not necessarily highlight areas in PM the identified publication discusses and gaps for further research. This is not necessarily an issue because project management is wide and applicable in different fields and has been identified in this articles as a limitation.

 Some information needed to be corrected includes the following:

 A. "2.6 -  Diffendal, (2021) Recent research from project management institute, dubbed “Pulse of the Profession®,” showed that more than eighty percent of participants indicated that AI influences their organization’s success. “Artificial Intelligence Entrepreneurs: Cracking the Code on Project Performance” suggests that project practitioners forestall an increase in the proportion of projects employing Artificial Intelligence from twenty-three percent to thirty-seven percent over the following three years of operation (Cockburn et al., 2018)".

 the first two lines of this paragraph is confusing. are you quoting Diffendal's writing on pulse of the profession, if so, the 'Recent' should start with a small letter. however, it is advisable to quote pulse of the profession directly for easier reference.

**Responses**

 We sincerely thank the reviewer for the constructive feedback on improving the literature review section 2.6 and the paragraph rewritten as thus:

 According to recent research from the project management institute, titled "Pulse of the Profession®," more than 80 percent of participants believe that AI influences the success of their firm. "Artificial Intelligence Entrepreneurs: Cracking the Code on Project Performance" recommends that project practitioners forestall an increase in the number of projects incorporating Artificial Intelligence from twenty-three percent to thirty-seven percent over the next three years of operation (Cockburn et al., 2018).

**Comment**

 "Figure 5 - This graphic presents a PRISMA flow diagram delineating the systematic review procedure for selecting studies. The figure adheres to a conventional format featuring alternating green and red boxes linked by arrows"

 **i see yellow and blue boxes** not purple and green.

**Responses**

 We thank the reviewer for his insight and necessary correction was made thus:

 The figure adheres to a conventional format featuring alternating light yellow and light blue boxes linked by arrows, illustrating the transition from initial identification to final inclusion.

**Comment**

 Figure 8 - the ppt slide is blurry and the only words I was able to deduce was Project management. It would be best to find a way to ensure that the figure is clear to your readers.  (I also downloaded the ppt and had the same issue)

 Figure 9 10 & 11 has same issue

**Responses**

 We thank the reviewer for the observation on Figures 8, 9, 10 and 11.

 We have generated another figures from the VOSviewer software and employed convert.town/image-dpi300 for the figures.

**Comment**

 Table 6 is a well detailed table; however it contains 13 papers, not 10 as stated. you can either remove 3 or change 10 to 13.

 We thank the reviewer for his insight and necessary correction was made as suggested:

 In the study of Brock & Von Wangenheim, (2019) they elucidate - replace off with by and elucidates with elucidate.

**Responses**

 We thank the reviewer for his insight and the correction was made “elucidate”.

 The authors declare that they have no known competing financial interests or personal relationships that could have appeared to influence the work reported in this paper.

# Reviewer report: Reviewer response for version 1

**DOI:** 10.5256/f1000research.187040.r461769

**Published:** 2026-03-09

**Authors:** Satyadhar Joshi

**Affiliations:** 1 Assistant Vice President, Global Analytics, Bank of America, Jersey City, NJ, USA

**S** **ummary of the Article**

 This study presents a mixed-methods investigation into the role of Artificial Intelligence (AI) in Project Management (PM). The authors aim to identify key challenges, strategies, and best practices for AI implementation. The scientometric analysis maps publication trends, keyword co-occurrence, influential institutions, countries, authors, and citation patterns. The qualitative review summarizes challenges and strategies for AI integration. The authors conclude that AI (ML, NLP, predictive analytics) can significantly enhance project efficiency, decision-making, and risk mitigation. Author should add a few lines about recent Agentic and Generative Systems just to be more current and relevant in todays lanscape.

**Evaluation and Detailed Comments** **with Recommendations for Revision**

 The dual-method approach is a useful, providing both a broad overview and a thematic synthesis. Below are a few low effort high impact ideas that can be addressed to improve the manuscript's clarity, rigor, and overall scientific contribution.

**2. Details of Methods and Analysis (Answer: Partly)**

 While the use of Scopus and VOSviewer is appropriate, several low effort fixes that will make the paper acceptable:

- **Search String** **Clearficaiton** **:** The final search string used is presented as [“Artificial Intelligence” OR “Project Management”] AND [“Challenges”] AND [“Strategies” OR “Best Practices”]. The authors can clarify the precise Boolean logic used and add in the paragagtph (e.g., TITLE-ABS-KEY ( ("artificial intelligence" OR "AI") AND ("project management") AND (challenges OR strategies OR "best practices") )). And then add which method was used to get to 293. This should be doable.
- **Qualitative Synthesis Methodology:** Section 3 on the qualitative analysis can be expanded and if there any excel author can add it on zenodo like author added other pdf. If not it can be accepted in current form.
- **Scientometric Thresholds:** The thresholds for inclusion in the scientometric analysis can be clearified by adding any issues with low number that author found. If not, it is acceptable in current form as well. This is a minor issue.

- **Keyword Analysis** **Diagrams** **:** The identification of clusters in the keyword co-occurrence map (Figure 8) can be explained in a few more lines. Adding line about the five distinct thematic clusters represented by the different colors can be done. Also Naming and discussing these clusters can be done. Just adding 2-3 lines will would provide significant insight into the intellectual structure of the field.

**4. Conclusions (Answer: Partly)**

 The conclusions adequately summarize the descriptive findings of the scientometric analysis (e.g., publication trends, top institutions). The author can add a line each on qualitative results and quantitative results. There is scope for adding 2-4 lines in conclusion.

- **Overly Broad** **Adjective fixes** **:** The conclusion that AI "significantly enhances project performance" can be changed to qualitative mentions. The qualitative section identified significant challenges (data quality, skills gap, resistance to change).

**Conclusion**

 This manuscript has the foundations of a valuable contribution to the field. With the recommended minor revisions, around methodology and interpretation, the paper could become a useful resource for researchers and practitioners in AI and project management.

Are the rationale for, and objectives of, the Systematic Review clearly stated?

Yes

Is the statistical analysis and its interpretation appropriate?

Partly

If this is a Living Systematic Review, is the ‘living’ method appropriate and is the search schedule clearly defined and justified? (‘Living Systematic Review’ or a variation of this term should be included in the title.)

Partly

Are sufficient details of the methods and analysis provided to allow replication by others?

Partly

Are the conclusions drawn adequately supported by the results presented in the review?

Partly

Reviewer Expertise:

Agentic AI, Generative AI, AI risk, AI Management, Risk Management, Business Management

I confirm that I have read this submission and believe that I have an appropriate level of expertise to confirm that it is of an acceptable scientific standard, however I have significant reservations, as outlined above.

# Response

**Published:** 2026-03-11

**Authors:** Akinlo Mogbojuri

**Affiliations:** Industrial Engineering, Durban University of Technology, Durban, KwaZulu-Natal, South Africa

**Reviewer 2**

 Summary of the Article

 This study presents a mixed-methods investigation into the role of Artificial Intelligence (AI) in Project Management (PM). The authors aim to identify key challenges, strategies, and best practices for AI implementation. The scientometric analysis maps publication trends, keyword co-occurrence, influential institutions, countries, authors, and citation patterns. The qualitative review summarizes challenges and strategies for AI integration. The authors conclude that AI (ML, NLP, predictive analytics) can significantly enhance project efficiency, decision-making, and risk mitigation. Author should add a few lines about recent Agentic and Generative Systems just to be more current and relevant in today’s landscape.

**We sincerely thank the reviewer for the constructive feedback on improving the summary of the article. We have added few lines to the abstract section about Agentic and Generative systems to make relevant in today’s landscape.**

 Evaluation and Detailed Comments with Recommendations for Revision

 The dual-method approach is a useful, providing both a broad overview and a thematic synthesis. Below are a few low effort high impact ideas that can be addressed to improve the manuscript's clarity, rigor, and overall scientific contribution.

**We thank the reviewer for the positive acknowledgment on the dual-method approach. The few low effort high impact ideas will be addressed to improve the manuscript.**

 2. Details of Methods and Analysis (Answer: Partly)

 While the use of Scopus and VOSviewer is appropriate, several low effort fixes that will make the paper acceptable:

 Search String Clearficaiton: The final search string used is presented as [“Artificial Intelligence” OR “Project Management”] AND [“Challenges”] AND [“Strategies” OR “Best Practices”]. The authors can clarify the precise Boolean logic used and add in the paragagtph (e.g., TITLE-ABS-KEY ( ("artificial intelligence" OR "AI") AND ("project management") AND (challenges OR strategies OR "best practices") )). And then add which method was used to get to 293. This should be doable.

**We sincerely appreciate your thorough review and constructive feedback regarding the details of methods and analysis. We have carefully addressed each of your concerns as detailed below.**

**Regarding the exact search queries with Boolean operators, we have now provided the complete verbatim search string in Section 3.1 (Search Technique and Retrieval of Literature). The exact query executed in Scopus is now explicitly stated as: TITLE-ABS-KEY Keywords ["Artificial Intelligence" OR "AI"] AND ["Project Management"] AND ["Challenges" OR "Strategies" OR "Best Practices"]. The keywords were carefully selected through expert consultation and preliminary scoping searches and employing Scopus search documents with the preliminary search produced 293 articles.**

 Qualitative Synthesis Methodology: Section 3 on the qualitative analysis can be expanded and if there any excel author can add it on zenodo like author added other pdf. If not it can be accepted in current form.

**We sincerely thank the reviewer for the constructive feedback on improving the qualitative analysis. We have substantially improved the qualitative analysis in section 3.3.**

 Scientometric Thresholds: The thresholds for inclusion in the scientometric analysis can be clearified by adding any issues with low number that author found. If not, it is acceptable in current form as well. This is a minor issue.

**We cited the Scientometric Thresholds in the result analysis section from 4.2 to 4.7**

 Keyword Analysis Diagrams: The identification of clusters in the keyword co-occurrence map (Figure 8) can be explained in a few more lines. Adding line about the five distinct thematic clusters represented by the different colors can be done. Also Naming and discussing these clusters can be done. Just adding 2-3 lines will would provide significant insight into the intellectual structure of the field.

**We have have identified the clusters in the keyword co-occurrence map in (Figure 8) with a few lines’ explanation including the five distinct thematic clusters representatives by the different colours.**

**Figure 8: The keyword co-occurrence map illustrates five distinct thematic clusters, each denoted by a unique hue, representing the intellectual substructures influencing research at the convergence of Artificial Intelligence and Project Management. The blue cluster emphasizes project management and underscores fundamental issues including digital transformation, decision-support systems, and managerial performance, reflecting a significant concentration on the integration of AI technology into essential PM processes. The red cluster focuses on project planning and operational optimization, incorporating terminology like scheduling, lean production, cost reduction, and critical path analysis, which highlights research on AI-enhanced planning efficiency and supply chain coordination. The green cluster encompasses the risk and decision analytics aspect of the domain, with terms like risk assessment, predictive models, and decision trees reflecting the increasing utilization of machine learning techniques for risk forecasting and quality control. The yellow cluster represents a perspective on technology adoption and construction management, linking artificial intelligence, information management, BIM, and applications within the construction industry, thereby demonstrating the integration of AI-enabled tools in project-based sectors like construction and infrastructure development. The purple cluster signifies the stream of knowledge and capability development, connecting project management education, software engineering, and management challenges, indicating a growing concern regarding the skills, learning systems, and organizational competencies necessary to implement AI in project settings. Collectively, these clusters indicate a domain that is concurrently advancing in operational optimization, risk intelligence, digital integration, sectoral applications, and capability development.**

 4. Conclusions (Answer: Partly)

 The conclusions adequately summarize the descriptive findings of the scientometric analysis (e.g., publication trends, top institutions). The author can add a line each on qualitative results and quantitative results. There is scope for adding 2-4 lines in conclusion.

**We have added few lines each on qualitative results and quantitative results in the conclusion section 5.1**

**Quantitative Results**

**The quantitative aspect of the study utilizes scientometric analysis of articles sourced from Scopus, concentrating on the development of Artificial Intelligence applications in project management research. The findings indicate a significant increase in academic productivity post-2018, along with the widespread adoption of machine learning and data-driven decision-making tools in engineering and management fields. Before this period, articles concerning AI in project management were infrequent and predominantly exploratory, typically emphasizing expert systems and rule-based decision models. The literature indicates a distinct transition towards data-intensive methodologies, including machine learning, natural language processing, and predictive analytics.**

**The quantitative results indicate that Artificial Intelligence research in project management is evolving from experimental methodologies to practical decision-support frameworks that can tackle intricate operational issues, including cost overruns, schedule delays, and resource allocation inefficiencies. This transition indicates a more comprehensive development within the discipline, wherein AI is regarded not just as an analytical instrument but increasingly as an integral element of project governance and operational intelligence frameworks.**

**Qualitative Results**

**The qualitative study consolidates ideas from the examined literature to pinpoint the primary difficulties, strategic approaches, and optimal practices related to the implementation of Artificial Intelligence in project management settings. Three principal thematic features arise from the interpretative review: organizational preparation, data governance and infrastructure, and human-AI collaboration in decision-making processes.**

**The qualitative findings indicate that the efficacy of Artificial Intelligence in project management is more contingent upon the overarching organizational and institutional contexts than on the complexity of the algorithms employed. Successful AI integration necessitates a synthesis of data maturity, governance alignment, and human-centric system design, ensuring that sophisticated analytical tools enhance rather than undermine established project management processes.**

**Acknowledgment to the Reviewer**

 We extend our deepest gratitude to the reviewer for taken valuable time, expertise, and constructive feedback on our manuscript. Your insightful comments have significantly enhanced the comprehensiveness, methodological rigor, clarity, and practical relevance of this review. The specific suggestions regarding summary of the study, literature coverage, methodology, future research directions, contribution and research gaps, and quantitative and qualitative results with broader AI in project management domains have been carefully addressed through substantial revisions. We believe that the collective wisdom reflected in your reviews has transformed this work into a more robust and impactful contribution to the AI in project management literature, and we are genuinely appreciative of your efforts in improving the quality of this research.

# Reviewer report: Reviewer response for version 1

**DOI:** 10.5256/f1000research.187040.r446009

**Published:** 2026-01-10

**Authors:** Nurkhat Ibadildin

**Affiliations:** 1 Astana IT University, Astana, Kazakhstan

1. Critical Analysis & Reviewer Comments

 Strengths

 Timeliness and Relevance: The integration of AI into Project Management (PM) is a highly relevant topic for both academia and industry. The manuscript addresses a significant gap by attempting to synthesize challenges and strategies.

 Methodological Framework:   The use of a hybrid approach—combining scientometric analysis (VOSviewer) with qualitative synthesis—is a sound choice for a systematic review.

 Visualization:   The PRISMA flow diagram and the bibliometric maps provide a clear overview of the data selection process and the current research landscape.

 Weaknesses & Technical Criticisms 

 Depth of Qualitative Synthesis:   While the scientometric section is detailed, the "Qualitative Analysis" (Section 3.3) remains largely descriptive. To meet academic standards, the "Best Practices" and "Strategies" sections need more critical depth rather than just listing previous studies (e.g., Section 2.5). There is a lack of a novel conceptual framework derived from these findings.

 Figure Redundancy and Chronology:   Figure 1 (Evolution of AI) ends at 2019, yet the study's scope extends to 2025. This creates a chronological disconnect. Furthermore, some figures (like Figure 1 and Figure 4) appear to be adapted from third-party sources (e.g., Alshaikhi, 2021; Stamford, 2019) without sufficient critical interpretation by the authors.

 Sample Size:   The systematic review is based on 59 papers. While focused, for a field as broad as AI and PM, the authors should justify why such a narrow set was selected and if significant regional or industry-specific journals were missed.

 Grammar and Syntax:   There are several typographical and phrasing issues (e.g., "The systematic review the implementation of challenges..." in the Abstract ). These must be corrected for a professional journal.

 Scientific Contribution:   The paper identifies  who  is writing and  what  keywords are used, but it does not sufficiently answer  how  specific AI technologies (like Generative AI or LLMs, which are current trends) specifically solve the PM challenges mentioned.

 2. Detailed Recommendations for Authors 

 1.   Enhance the Discussion:   Move beyond bibliometric data. Explain  why  certain countries (like China and Morocco) are leading and what specific project management methodologies (Agile vs. Waterfall) are most impacted by AI according to the literature.

 2. Update "Best Practices":   The current "Best Practices" section (2.5) cites medical and clinical AI studies (radiotherapy, radiology). While cross-disciplinary learning is good, the authors must bridge these findings more explicitly to the context of Project Management.

 3.   Refine the Research Gap:   Explicitly state what your review offers that existing reviews on "AI in PM" do not.

 4.   Language Edit:   A thorough professional English language edit is required to ensure the manuscript meets the high linguistic standards of Elsevier.

 3. Decision: Major Revision 

 Justification: 

 The manuscript presents a valuable bibliometric overview of the AI-PM landscape and follows a structured PRISMA methodology. However, the qualitative synthesis lacks the "Best Practices" depth promised in the title. The connection between the bibliometric clusters and the practical strategies is currently weak. Once the authors deepen the thematic analysis and address the chronological gaps in their literature evolution, this paper has the potential to be a high-impact contribution to the field.

Are the rationale for, and objectives of, the Systematic Review clearly stated?

Yes

Is the statistical analysis and its interpretation appropriate?

Yes

If this is a Living Systematic Review, is the ‘living’ method appropriate and is the search schedule clearly defined and justified? (‘Living Systematic Review’ or a variation of this term should be included in the title.)

Partly

Are sufficient details of the methods and analysis provided to allow replication by others?

Yes

Are the conclusions drawn adequately supported by the results presented in the review?

Yes

Reviewer Expertise:

AI in Project Management

I confirm that I have read this submission and believe that I have an appropriate level of expertise to confirm that it is of an acceptable scientific standard, however I have significant reservations, as outlined above.

# Response

**Published:** 2026-03-11

**Authors:** Akinlo Mogbojuri

**Affiliations:** Industrial Engineering, Durban University of Technology, Durban, KwaZulu-Natal, South Africa

**Reviewer #1:**

 1. Critical Analysis & Reviewer Comments

 Strengths

 Timeliness and Relevance: The integration of AI into Project Management (PM) is a highly relevant topic for both academia and industry. The manuscript addresses a significant gap by attempting to synthesize challenges and strategies.

 Methodological Framework:   The use of a hybrid approach combining scientometric analysis (VOSviewer) with qualitative synthesis is a sound choice for a systematic review.

 Visualization:   The PRISMA flow diagram and the bibliometric maps provide a clear overview of the data selection process and the current research landscape.

**Authors response #1** **:**

 We thank the reviewer for the positive acknowledgment of our manuscript timelines and relevance, methodological framework, and visualization.

 Weaknesses & Technical Criticisms

 Depth of Qualitative Synthesis:   While the scientometric section is detailed, the "Qualitative Analysis" (Section 3.3) remains largely descriptive. To meet academic standards, the "Best Practices" and "Strategies" sections need more critical depth rather than just listing previous studies (e.g., Section 2.5). There is a lack of a novel conceptual framework derived from these findings.

 Figure Redundancy and Chronology:   Figure 1 (Evolution of AI) ends at 2019, yet the study's scope extends to 2025. This creates a chronological disconnect. Furthermore, some figures (like Figure 1 and Figure 4) appear to be adapted from third-party sources (e.g., Alshaikhi, 2021; Stamford, 2019) without sufficient critical interpretation by the authors.

 Sample Size:   The systematic review is based on 59 papers. While focused, for a field as broad as AI and PM, the authors should justify why such a narrow set was selected and if significant regional or industry-specific journals were missed.

 Grammar and Syntax:   There are several typographical and phrasing issues (e.g., "The systematic review the implementation of challenges..." in the Abstract). These must be corrected for a professional journal.

 Scientific Contribution:   The paper identifies who is writing and  what  keywords are used, but it does not sufficiently answer  how  specific AI technologies (like Generative AI or LLMs, which are current trends) specifically solve the PM challenges mentioned.

**Authors response #2:**

**Depth of Qualitative Synthesis:**

 We sincerely thank the reviewer for the constructive feedback on improving the qualitative analysis. We have substantially improved the qualitative analysis in section 3.3.

 We have substantially expanded the best practices of AI in more in-depth as suggested by the reviewer in section 2.5.

**Figure Redundancy and Chronology:**

 We have extended the evolution of AI from 2007 to 2025 with the study’s scope in Figure 1.

 We have explained and interpreted the figure 1 and figure 4 as thus:

**Fig 1:** The evolution of artificial intelligence from 2007 to 2025 highlights significant milestones in Early machine learning & foundational tools, Deep learning revolution (AlexNet),  iphone & Mobile AI, google predictions, imageNet launch, deep learning era, word2vec, AlphaGo, OpenAI, GANS, Language models revolutionize NLP, GPT-2 Released, Multimodal AI, AI video & Agents and AI reasoning models, AI Governance & Global Summits  featuring notable figures and companies such as Siri Precursor Technology, Computer Vision Dataset Revolution, TenserFlow, Claude Sonnet, Sora Claude, Mainstream Claude Gemini LLama, Alan Turing, Newell, GE, Ian Horswill, Apple, Google, and Microsoft.

 Figure 4: The facets of PM that potentially benefit from AI aid are shown by each bar, which delineates the specific area of PM deemed appropriate for AI assistance based on several survey results. The elevation of each bar directly correlates with the answer tally on the y-axis.

**Sample Size:**  

 We also justified the narrow on papers selected based on the scope of the topic “Artificial Intelligence in Project Management” not “application of Artificial Intelligence in Project Management”

**Grammar and Syntax:**

 We have corrected the typographical and phrasing issues as suggested.

**Scientific Contribution:**  

 We have specified how some AI technologies solved the PM in the conclusion section 5.1

 The recent advancements in Artificial Intelligence, especially Generative AI and Large Language Models, have revolutionized project management by tackling persistent issues associated with interactions, anticipating, decision-making uncertainty, and knowledge governance. Predictive machine learning enhances the timeline and price precision, whereas generative models automate documentation and enable real-time information retrieval. Reinforcement learning and optimization algorithms facilitate adaptive resource allocation amidst uncertainty, while natural language processing and computer vision improve stakeholder involvement and progress assessment. These AI tools collectively transition project management from reactive oversight to innovative, data-informed, and intelligent choice-making.

 2. Detailed Recommendations for Authors

 1.   Enhance the Discussion:   Move beyond bibliometric data. Explain  why  certain countries (like China and Morocco) are leading and what specific project management methodologies (Agile vs. Waterfall) are most impacted by AI according to the literature.

 2. Update "Best Practices":   The current "Best Practices" section (2.5) cites medical and clinical AI studies (radiotherapy, radiology). While cross-disciplinary learning is good, the authors must bridge these findings more explicitly to the context of Project Management.

 3.   Refine the Research Gap:   Explicitly state what your review offers that existing reviews on "AI in PM" do not.

 4.   Language Edit:   A thorough professional English language edit is required to ensure the manuscript meets the high linguistic standards of Elsevier.

**Authors response #3:**

**1.   Enhance the Discussion:** 

 We have explained why countries like China, Morocco and USA are leading in results and discussion section 4.4 as thus:

 The importance of nations like the United States, China, and Morocco in AI-driven project management research partnerships illustrates disparities in research capability, strategic investment, and collaborative behavior. The United States operates as a global center of knowledge owing to its multidisciplinary academic framework. China's influence is propelled by substantial research output and a robust convergence between national AI policies and infrastructure-centric project management applications. Conversely, Morocco acts as a pivotal intermediary nation, utilizing specific research issues and broad international partnerships to link African, European, and worldwide research groups. Collectively, these trends elucidate their significance in the scientometric network mapping at the national level. Regarding research efficiency, China and Morocco are the most prolific nations

**2. “Update "Best Practices":**

 We have updated the best practices in AI implementation in the literature review section 2.5

**3.  Refine the Research Gap:**

 We have refined the research gap in the conclusion section 5.1

 This review enhances the literature on AI in Project Management by transitioning from technology-centric descriptions to a comprehensive framework encompassing challenges, strategies, and best practices. This study specifically associates implementation difficulties with actual mitigation measures and empirically validated best practices, in contrast to prior evaluations that only enumerate AI tools or applications. This paper is one of the initial analyses to comprehensively integrate Generative AI with Large Language Models, emphasizing their revolutionary impact on communication, information management, and decision assistance. The review provides practical counsel for practitioners, organizational leaders, and policymakers

**4.   Language Edit:**

 We have carried a proofreading to enhance the manuscript.
